# Dual activities of the anti-cancer drug candidate PBI-05204 provide neuroprotection in brain slice models for neurodegenerative diseases and stroke

**DOI:** 10.1038/srep25626

**Published:** 2016-05-12

**Authors:** Michael J. Van Kanegan, Denise E. Dunn, Linda S. Kaltenbach, Bijal Shah, Dong Ning He, Daniel D. McCoy, Peiying Yang, Jiangnan Peng, Li Shen, Lin Du, Robert H. Cichewicz, Robert A. Newman, Donald C. Lo

**Affiliations:** 1 Center for Drug Discovery and Department of Neurobiology, Duke University Medical Center, Durham, NC 27710, US; 2 Department of Palliative Care, Rehabilitation and Integrative Medicine, University of Texas M. D. Anderson Cancer Center, Houston, TX 77030, US; 3 Department of Chemistry and Biochemistry, University of North Carolina at Wilmington, NC 28403, US; 4 Department of Pharmacy, Medical College of Yangzhou University, Yangzhou, China; 5 Department of Chemistry and Biochemistry, Stephenson Life Sciences Research Center, Natural Products Discovery Group, and Institute for Natural Products Applications and Research Technologies, University of Oklahoma, Norman, Oklahoma 73019, US; 6 Department of Experimental Therapeutics, University of Texas, M. D. Anderson Cancer Center, Houston, TX 77030, US.

## Abstract

We previously reported neuroprotective activity of the botanical anti-cancer drug candidate PBI-05204, a supercritical CO_2_ extract of *Nerium oleander*, in brain slice and *in vivo* models of ischemic stroke. We showed that one component of this neuroprotective activity is mediated through its principal cardiac glycoside constituent, oleandrin, via induction of the potent neurotrophic factor brain-derived neurotrophic factor (BDNF). However, we also noted that the concentration-relation for PBI-05204 in the brain slice oxygen-glucose deprivation (OGD) model is considerably broader than that for oleandrin as a single agent. We thus surmised that PBI-05204 contains an additional neuroprotective component(s), distinct from oleandrin. We report here that neuroprotective activity is also provided by the triterpenoid constituents of PBI-05204, notably oleanolic acid. We demonstrate that a sub-fraction of PBI-05204 (Fraction 0–4) containing oleanolic and other triterpenoids, but without cardiac glycosides, induces the expression of cellular antioxidant gene transcription programs regulated through antioxidant transcriptional response elements (AREs). Finally, we show that Fraction 0–4 provides broad neuroprotection in organotypic brain slice models for neurodegeneration driven by amyloid precursor protein (APP) and tau implicated in Alzheimer’s disease and frontotemporal dementias, respectively, in addition to ischemic injury modeled by OGD.

Common signaling pathways and therapeutic opportunities relating cancer and neurodegeneration have become increasingly apparent in recent years^1^,^2^. Numerous cellular processes and potential intervention points long thought to be uniquely relevant to oncology are now finding potential applications in CNS neurodegeneration and injury, and vice versa. For example, the FDA-approved anti-cancer drug bexarotene was reported to provide benefit in animal models of Alzheimer’s disease (AD), and is currently being investigated in human clinical trials^3^. Conversely, roles for neurotransmitters and neurotrophic factors in tumor progression and reprogramming of the stromal microenvironment are increasingly appreciated^4^,^5^.

In this context, we previously reported the neuroprotective activity of the anti-cancer botanical drug candidate PBI-05204^6^ in brain tissue and *in vivo* models for ischemic stroke^7^. We showed that an essential neuroprotective constituent of PBI-05204, a supercritical CO_2_ extract of *Nerium oleander*, was the cardiac glycoside oleandrin. Unlike the approved cardiac glycoside drugs digoxin and digitoxin, oleandrin is blood-brain barrier (BBB) penetrant^7^. We subsequently demonstrated that the neuroprotective activity of the oleandrin component of PBI-05204 is mediated by induction of neural expression of the potent CNS neurotrophic factor brain-derived neurotrophic factor (BDNF)^8^.

However, we noted that the concentration-response relation for PBI-05204 in the brain-slice OGD model is broad and cannot be fully accounted for by the relatively narrower concentration-response relation for oleandrin as a single agent (see Fig. 4B in^7^). We thus hypothesized the existence of an additional neuroprotective constituent(s) of PBI-05204 that affords neuroprotection through an independent mechanism. We provide evidence here that this additional neuroprotective component of PBI-05204 is the triterpenoid oleanolic acid, with an additional potential contribution from the closely related triterpene ursolic acid. We demonstrate general neuroprotective activity of a subfraction of PBI-05204 containing oleanolic and ursolic acids, termed PBI-04711, in additional models for CNS neurodegeneration. Finally, we demonstrate the programmatic induction of nuclear factor erythroid 2 related factor 2 (Nrf2)-dependent antioxidant genes as a potential mechanism underlying the neuroprotective activity of PBI-04711 and its triterpenoid components.

## Results

### Identification of neuroprotective subfractions of PBI-05204

We previously reported robust neuroprotection in the brain slice OGD model provided by the full PBI-05204 supercritical CO_2_ extract of *Nerium oleander* across a broad range of concentrations, whereas the concentration-response relation for its principal cardiac glycoside component, oleandrin, was much narrower and thereby could likely account quantitatively for only a portion of the neuroprotective activity of the full botanical extract (see Fig. 4B in^7^). As PBI-05204 likely contains many pharmacologically active phytochemicals in addition to oleandrin, we used a chemical fractionation schema (see Methods) to separate the PBI-05204 extract into a series of 5 subfractions. These subfractions were characterized as containing or not containing oleandrin and/or other cardiac glycoside components, as summarized in Table 1 and described in detail in the Methods.

**Figure 1:**
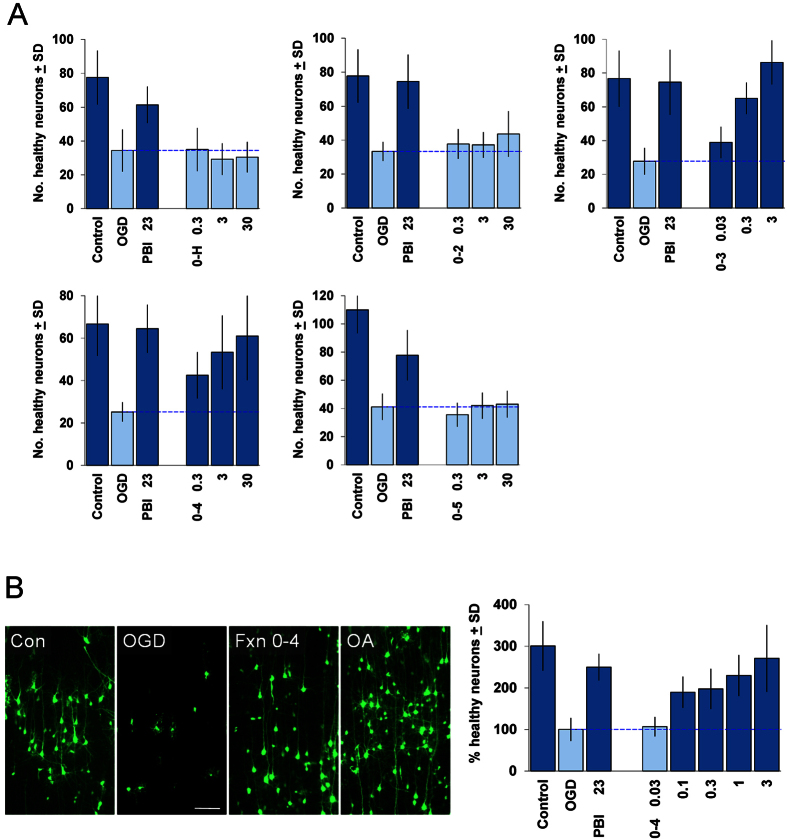
(**A**) Evaluation of subfractions of PBI-05204 in the brain slice oxygen-glucose deprivation (OGD) assay. Coronal brain slice explants were prepared and subjected to 5.5 min. transient OGD as previously described^7^ (see Methods for more detailed description). Numbers of healthy cortical pyramidal neurons in the each brain slice were scored 24 h later. The first 3 bars in each graph show: control brain slices not subjected to OGD (“Control”); negative-control brain slices subjected to OGD and treated with DMSO carrier only (“OGD”); and positive-control brain slices subjected to OGD and treated with 23 μg/ml of the full PBI-05204 extract (“PBI 23”). Subfractions were tested at the concentrations indicated in units of μg/ml. Only Fractions 0–3 and 0–4 provided significant neuroprotection at the concentrations tested (concentrations of Fraction 0–3 of 10 μg/ml and above exhibited toxicity). (**B**) Confirmation of the neuroprotective activity of Fraction 0–4. *Left,* example photomicrographs showing: cortical neurons expressing YFP in a control brain slice (“Con”); and in brain slices subjected to OGD and treated with DMSO-carrier only (“OGD”), 3 μg/ml Fraction 0–4 (“Fxn 0–4”), or 10 μM oleanolic acid (“OA”). *Right,* Concentration-response relation for Fraction 0–4 in the brain slice OGD assay, in units of μg/ml; the average of 3 independent experiments is shown, with the OGD negative-control condition set to 100%. For all graphs, dark blue bars denote statistically significant differences with respect to the OGD negative-control by ANOVA followed by Dunnett’s *post hoc* comparison test at the 0.05 confidence level.

We then evaluated each of the subfractions of PBI-05204 in the brain slice OGD assay. As shown in Fig. 1A, only 2 of these 5 subfractions showed significant neuroprotection: Fraction 0–3, which contained both oleandrin as well as other cardiac glycoside constituents; and Fraction 0–4, which was devoid of any cardiac glycoside constituents. Fraction 0–4 nevertheless provided strong neuroprotection in the brain slice OGD assay to levels comparable to that for the full PBI-05204 extract, and did so in a concentration-dependent manner (Fig. 1B).

### Fraction 0–4 provides broad neuroprotection in brain slice neurodegeneration models

**Figure 2:**
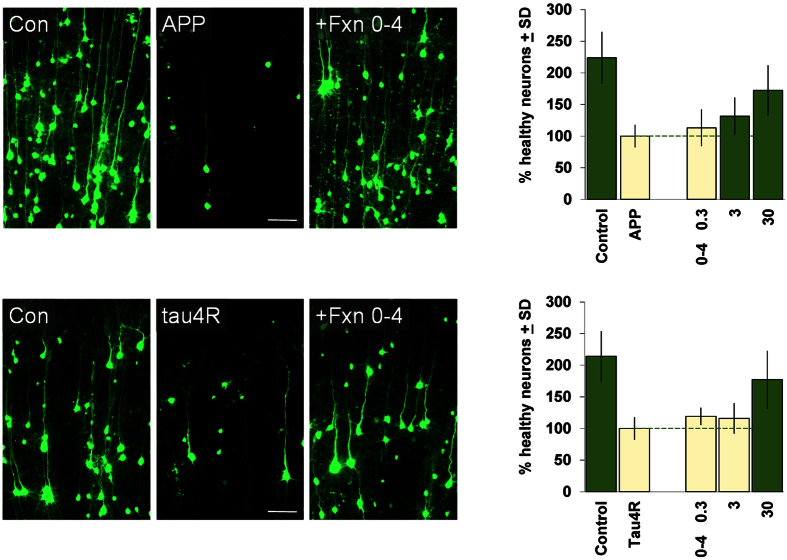
Neuroprotective activity of Fraction 0–4 in brain slice assays for neurodegeneration induced by APP and tau. *Left,* example photomicrographs showing cortical neurons transfected with YFP only (“Con”) or with YFP plus a human WT amyloid precursor protein (“APP”) or a human tau4R0N (“tau4R”) expression construct as indicated. Overt neurodegeneration and cell loss driven by either APP or tau4R by 3 days after transfection (compare middle to left panels) could be rescued by treatment with 30 μM Fraction 0–4 (right panels). *Right,* Concentration-response relations for Fraction 0–4 in the brain slice APP and tau4R assays as indicated. Averages of 3 and 4 independent runs are shown for APP and tau4R, respectively, with the negative-control conditions (treated with DMSO only) set to 100%. For both graphs, dark green bars denote statistically significant differences with respect to the respective APP or tau4R negative-controls by ANOVA followed by Dunnett’s *post hoc* comparison test at the 0.05 confidence level.

We next asked if the neuroprotective activity of Fraction 0–4 was specific for neuronal injury induced by ischemia as modeled by OGD in the brain slice model, or if it could also provide neuroprotection in other neurodegenerative contexts. In fact, we found that Fraction 0–4 could also provide strong neuroprotection in 2 additional brain slice models in which cortical neuronal degeneration is driven by biolistic transfection of expression constructs for genes implicated in CNS neurodegeneration, namely, amyloid precursor protein (APP) and tau. In these models, APP and tau transfection induces progressive neurodegeneration of cortical neurons over the course of 3–4 days, in contrast to the neuronal injury and death caused by OGD which occurs over a 24 h period in the brain slice model^9^,^10^,^11^. As shown in Fig. 2, Fraction 0–4 provided significant concentration-dependent neuroprotection in both the APP and tau brain slice neurodegeneration models, albeit in a somewhat higher concentration range compared to that observed for OGD (c.f. Fig. 1B). As Fraction 0–4 was provided only as a single-bolus administration at the beginning of these longer-term assays, the apparent right-shift in concentration-response could have been due to compound turnover in the intact brain tissue environment of these assays.

### The principal constituents of Fraction 0–4 are triterpenoids, of which oleanolic acid is the most abundant

We next sought to determine the chemical composition of Fraction 0–4. Further chemical separations followed by 1D and 2D NMR spectroscopy showed that Fraction 0–4 was composed mostly of triterpenoids, of which oleanolic acid was the principal constituent (~35% by mass), followed by the closely related triterpenoids ursolic acid and betulinic acid (~25% and ~11%, respectively; see Methods and Supplementary Fig. S1). Additional minor components detected were also related triterpenes, all at <1% relative abundance: uvalol, ursolaldehyde (ursolic aldehyde), and 3*β*,27-dihydroxy-12-ursen-28-oic acid, with the exception of *E*/*Z*-27-(*p*-coumaroyl)ursolic acid which was present at ~2%.

**Figure 3:**
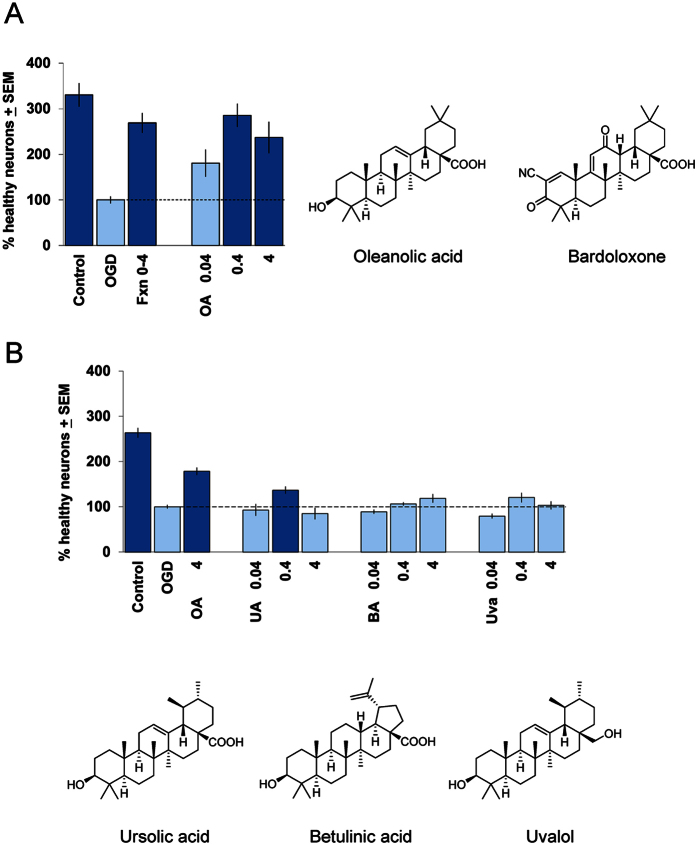
Neuroprotective activities of oleanolic acid and other major triterpenoid constituents of Fraction 0–4 evaluated as single agents. (**A**) Concentration-response relation for oleanolic acid (OA; in μg/ml) in the brain slice OGD assay (*left*); the average of 4 independent experiments is shown, with the OGD negative-control condition set to 100%. The positive control was 10 μg/ml Fraction 0–4 (“Fxn 0–4”). Dark blue bars denote statistically significant differences with respect to the OGD negative control by ANOVA followed by Dunnett’s *post hoc* comparison test at the 0.05 confidence level. *Right,* Comparison of the chemical structures of the triterpenoids oleanolic acid and bardoxolone. (**B**) Potential contributions to neuroprotection by additional triterpenoid constituents of Fraction 0–4. Concentration-response relations for ursolic acid (UA), betulinic acid (BA), and uvalol (Uva; all in μg/ml) in the brain slice OGD assay are shown (*above*). Averages for 2 independent experiments are included for each compound, with the OGD negative-control condition scaled to 100% and data plotted on the same axes for ease of comparison. The positive control was 4 μg/ml oleanolic acid (OA). Note that these are equimolar concentrations for each compound as the molecular weights for all are identical except for uvalol which was tested at 0.039, 0.39, and 3.88 μg/ml rounded to a single significant digit for display purposes. Dark blue bars denote statistically significant differences with respect to the OGD negative control by ANOVA followed by Dunnett’s *post hoc* comparison test at the 0.05 confidence level. *Below,* Comparison of chemical structures of the additional triterpenoid constituents of Fraction 0–4, ursolic acid, betulinic acid, and uvalol, as indicated.

To ask if the most abundant triterpenoid in Fraction 0–4, oleanolic acid (35%), itself could account for the neuroprotective activity of Fraction 0–4, we next tested this molecular constituent as a single agent and found, in fact, that a pure preparation of oleanolic acid could also provide robust and concentration-dependent neuroprotection in the brain slice OGD assay (Figs 1B and 3A). Interestingly, 0.4 μg/ml oleanolic acid provided near-complete rescue against neuronal injury induced by OGD, corresponding to its fractional activity in ~1 μg/ml of Fraction 0–4 which in Fig. 1B shows a comparable level of neuroprotection.

### Contribution from additional triterpenoid components of Fraction 0–4

Next, we evaluated the other 2 significant triterpenoid components of Fraction 0–4, ursolic acid (~25%) and betulinic acid (11%) as single agents in the OGD brain slice assay. For comparison, we also evaluated uvalol (0.4%) which among the minor triterpenoid component of Fraction 0–4 was the only one commercially available. We found that ursolic acid could provide minor but significant neuroprotection in the OGD brain slice assay, whereas neither betulinic acid nor uvalol could provide significant neuroprotection in the same equimolar concentration range (Fig. 3B). Thus, we concluded that oleanolic acid, as a single agent, provided the most robust neuroprotection among the principal triterpenoid constituents of Fraction 0–4 and could account for the majority of its neuroprotective effects.

### Activation of Nrf2-mediated ARE antioxidant gene pattern responses

Oleanolic acid is in a class of triterpenoids typified by compounds such as bardoxolone (Fig. 3A, *right*) which have been shown to be potent activators of the innate cellular phase 2 detoxifying pathway, in which activation of the transcription factor Nrf2 leads to transcriptional increases in programs of downstream antioxidant genes containing the antioxidant transcriptional response element (ARE)^12^,^13^. Bardoxolone itself has been extensively investigated in clinical trials in inflammatory conditions; however, a Phase 3 clinical trial in chronic kidney disease was terminated due to adverse events that may have been related to known cellular toxicities of certain triterpenoids including bardoxolone at elevated concentrations^14^,^15^.

**Figure 4:**
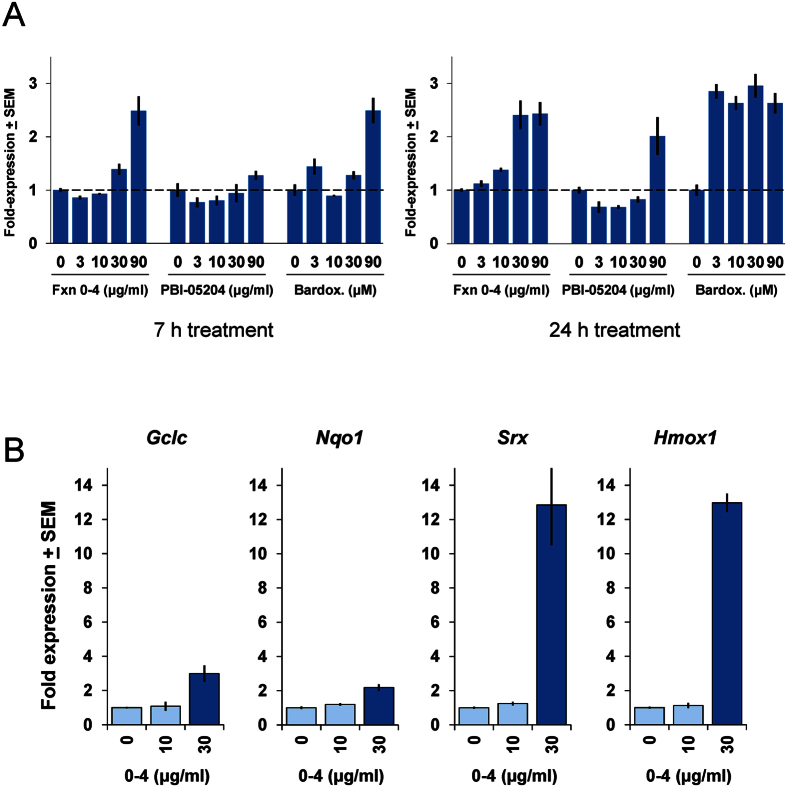
Activation of Nrf2/ARE antioxidant response pathways by Fraction 0–4. (**A**) Concentration- and time-dependent induction of a 5×-ARE-luciferase transcriptional reporter by Fraction 0–4, PBI-05204, and bardoxolone in mouse primary corticostriatal neuronal co-cultures. Activation of the 5×-ARE luciferase reporter became stronger with an apparent left-shift in concentration-response for extended treatment over 24 h (right graphs) compared to 7 h (left graphs). Fold-expression changes are expressed relative to the DMSO-carrier only condition (“0”) normalized to a co-transfected, constitutive *Renilla* luciferase control and set to a value of 1. (**B**) Fraction 0–4 induces robust upregulation of canonical ARE target genes, shown here for glutamate-cysteine ligase, catalytic subunit (*Gclc*); NAD(P)H:quinone oxidoreductase 1 (*Nqo1*); sulfiredoxin antioxidant protein (*Srx*); and heme oxygenase 1 (*Hmox1*)). Primary mouse corticostriatal co-cultures were treated with Fraction 0–4 at the concentrations indicated for 6 h, then harvested and processed for qPCR analysis of the ARE target genes shown. Quantitative RNA values are normalized to the GAPDH reference control and fold-expression changes are expressed relative to the DMSO-carrier only condition (“0”) set to a value of 1.

In contrast, Fraction 0–4 and oleanolic acid have not exhibited observable cellular toxicity in our hands and provide robust neuroprotection in the brain slice OGD model (Figs. 1B and 3A). We thus asked if Fraction 0–4 and oleanolic acid could be shown to activate the Nrf2-ARE gene pathway in neurons, using a corticostriatal primary neuronal co-culture system composed of the same neuronal and glial cell types represented in our brain slice assays^16^,^17^,^18^. First, we introduced into neuronal co-cultures an ARE-luciferase promoter-reporter construct which has been used extensively as an assay for Nrf2 activation^19^. As can be seen in Fig. 4A, treatment with Fraction 0–4 led to clear, concentration- and time-dependent increases in activation of the ARE-luciferase reporter, to levels similar to that induced by the reference triterpenoid bardoxolone. By 24 h of treatment, significant induction of the ARE-luciferase reporter was seen in similar concentration ranges that provided neuroprotection in the OGD, APP, and tau brain slice neuroprotection assays (compare to Figs. 1B and 2). The full PBI-05204 extract increased ARE-luciferase transcription only at the highest concentration tested, presumably reflecting lower relative abundance of its triterpenoid constituents including oleanolic acid.

Next, we asked if endogenous ARE target gene transcription could also be shown to be induced by treatment with Fraction 0–4. In fact, as can be seen in Fig. 4B, the 4 canonical targets of Nrf2 activation assayed (the ARE genes glutamate-cysteine ligase, catalytic subunit (*Gclc*); NAD(P)H:quinone oxidoreductase 1 (*Nqo 1*); sulfiredoxin antioxidant protein (*Srx*); and heme oxygenase 1 (*Hmox1*)), were all significantly increased by treatment with Fraction 0–4 in a concentration-dependent manner, and to levels comparable to that induced by other known Nrf2 activators such as dimethyl fumarate (DMF; data not shown).

**Figure 5:**
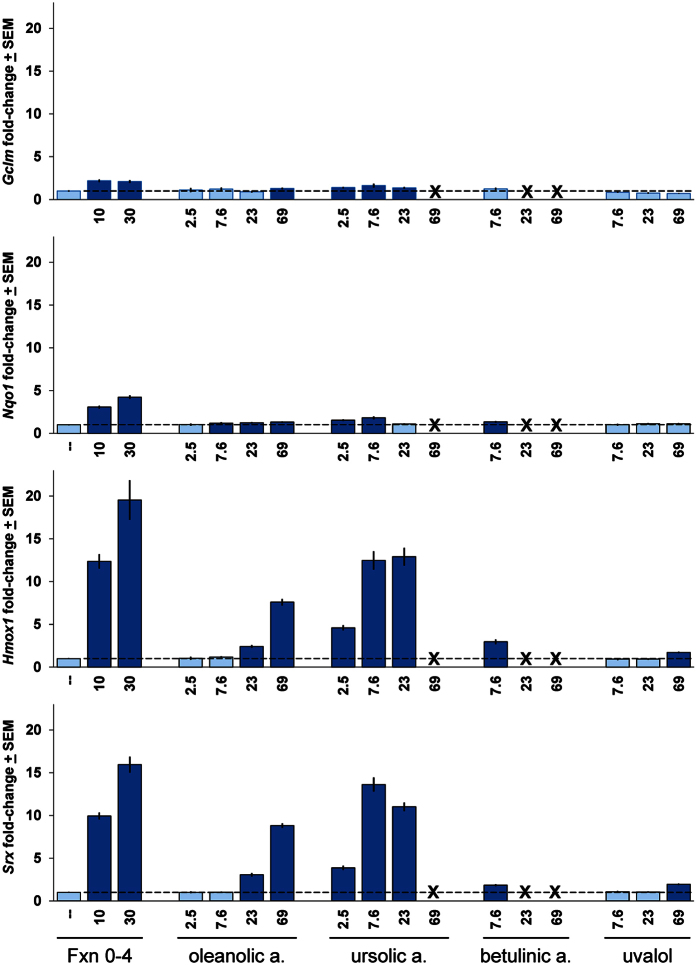
Activation of Nrf2/ARE antioxidant response pathways by individual triterpenoid constituents of Fraction 0–4. Oleanolic acid and ursolic acid induced robust upregulation of canonical ARE target genes to similar extents compared to Fraction 0–4, and in a strikingly similar pattern, shown here for glutamate-cysteine ligase, modifier subunit (*Gclm*); *Nqo1, Srx,* and *Hmox1.* Betulinic acid was also able to induce ARE gene expression in an intermediate concentration between 7.6 and 23 μg/ml despite its toxicity (see Supplementary Fig. S2). By comparison, uvalol was not able to induce ARE gene expression to a notable extent at any concentration tested. “X” symbols denote concentrations of compounds which induced toxicity and for which recovery of residual mRNA was insufficient to support qPCR analysis. Rat primary corticostriatal co-cultures were treated for 6 h with Fraction 0–4 (in μg/ml) or oleanolic acid, ursolic acid, betulinic acid, or uvalol (all in μM) at the concentrations indicated, then harvested and processed for qPCR analysis of the ARE target genes shown. Quantitative RNA values were normalized to the *GAPDH* reference control and fold-expression changes are expressed relative to the DMSO-carrier only condition (“–”) set to a value of 1. Dark blue bars denote statistically significant differences with respect to the DMSO-carrier only control by a Student’s *t*-test at p < 0.05.

Finally, we asked if the triterpenoid components of Fraction 0–4 could account for its activation of the Nrf2/ARE antioxidant gene pathway. Using ARE target gene activation as described above, we found that both oleanolic acid and ursolic acid induced substantial induction of the ARE target genes *Hmox1* and *Srx* and to a much lesser but significant extent *Nqo1* and *Gclm* (Fig. 5). This pattern of ARE gene induction was strikingly similar to that induced by the parental Fraction 0–4 (c.f. Fig. 4B). Curiously, ursolic acid appeared to be more potent than oleanolic acid in inducing ARE target gene expression. However, ursolic acid and betulinic acid also developed considerable toxicity at higher concentrations, which likely limit their net contributions to neuroprotection (see also Supplementary Fig. S2). Uvalol did not induce notable levels of ARE gene expression at any concentration tested.

## Discussion

While much progress has been made in determining genetic and epigenetic risk factors for ischemic stroke and neurodegenerative diseases including AD and frontotemporal dementias (FTDs), there remains an urgent need to identify drug targets and pathways that can provide direct neuroprotection to injured and degenerating neurons in these disease areas^20^,^21^,^22^,^23^. In recent years, promising sources for such candidate therapeutic targets and interventions have been unexpectedly emerging from oncology studies^1^,^2^.

In this context, we have identified a second component of the botanical anti-cancer drug candidate PBI-05204 that provides robust neuroprotection in multiple brain slice models for neuronal injury and neurodegeneration. PBI-05204 has been through a Phase I clinical trial^6^ and is currently in a Phase II trial for patients with advanced pancreatic cancer. We initially had reported neuroprotective activity of PBI-05204 mediated via its cardiac glycoside constituent oleandrin^7^. The mechanism of neuroprotective action of oleandrin transpired to be through endogenous neural induction of the potent neurotrophic factor BDNF^8^. In fact, we had previously identified the neuroprotective activity of cardiac glycosides in an hypothesis-neutral, drug-repositioning screen using a brain slice-based, high-throughput biology assay in which neuronal injury was induced by transient OGD, subsequently providing *in vivo* validation in two independent whole-animal models for focal ischemia^24^.

Interestingly, the novel Fraction 0–4 component of PBI-05204 we report here is devoid of any cardiac glycoside constituents including oleandrin. Rather, we have provided evidence that the most neuroprotective molecular constituent of Fraction 0–4 is the triterpenoid oleanolic acid, suggesting that the neuroprotective activity of Fraction 0–4 may be mediated through the known antioxidant, anti-inflammatory, and neuroprotective actions of this class of triterpenoids^25^,^26^,^27^,^28^. In addition, we found additional, though modest, contribution to neuroprotection from the next most abundant constituent of Fraction 0–4, the closely related triterpenoid ursolic acid. In fact, ursolic acid has previously been reported to provide neuroprotection in mouse models for cerebral ischemia and subarachnoid hemorrhage^29^,^30^.

Consistent with this idea, we showed that Fraction 0–4 induced clear activation of the Nrf2 transcription factor as well as increased expression of canonical downstream ARE target genes in concentration ranges overlapping with those that provided neuroprotection in brain slice models for stroke and neurodegenerative disease. Such ARE gene activation was mimicked by the most abundant triterpenoid constituents of Fraction 0–4, oleanolic acid, ursolic acid and betulinic acid, when tested as single agents. Although ursolic acid appeared to be more potent than oleanolic acid in ARE gene induction, ursolic acid (as well as betulinic acid) also developed considerable toxicity at higher concentrations which was not observed for oleanolic acid (Fig. 5). It is thus possible that such toxicity limits the net contribution of ursolic acid and betulinic acid to neuroprotection in the OGD brain slice assay despite their innate ability to induce ARE gene expression (see also Supplementary Fig. S2).

Neuroinflammation is increasingly appreciated to accompany not only acute CNS injury but also a wide range of chronic neurodegenerative conditions including Huntington’s disease and Alzheimer’s disease^31^,^32^,^33^,^34^. In many cases, there is evidence that such neuroinflammation amplifies/exacerbates neuronal stress; thus, anti-inflammatory strategies are actively being investigated in the treatment of a number of neurodegenerative conditions including through the induction of Nrf2-mediated innate antioxidant response networks^35^,^36^,^37^. An exciting advance in this area was the recent FDA-approval of the Nrf2 activator DMF (tradename Tecfidera; Biogen Idec) for the treatment of relapsing multiple sclerosis^38^,^39^.

We thus suggest that neuroprotection provided by the botanical drug candidate PBI-05204 across a surprisingly broad range of concentrations^7^ can be explained by the combined action of two mechanistically distinct pathways: one direct through neuroprotection mediated by induction of BDNF expression by its cardiac glycoside constituents^8^, while the other potentially indirect through mobilization of Nrf2-dependent antioxidant gene expression programs induced by its triterpenoid constituents as reported here. As such, our findings suggest that Fraction 0–4 itself (also termed PBI-04711) may have therapeutically relevant neuroprotective potential, independent of the parental PBI-05204 mixture, mediated by its anti-inflammatory activity across a number of neurodegenerative disorders including stroke, Alzheimer’s disease, and frontotemporal dementias.

The value of polypharmacology is increasingly appreciated in a range of disease areas^40^,^41^, for which natural products are a continuing resource for addressing known and emerging targets of therapeutic relevance, including in the CNS^42^,^43^. Natural product mixtures such as PBI-05204 may thus merit further investigation as multimodal drug candidates in addition to synthetic strategies to combine multiple target activities into a single small molecule drug^41^. Intriguingly, both the cardiac glycoside activity of PBI-05204 that we have previously reported^7^,^8^ as well as the triterpenoid-mediated, Nrf2-inducing activity we report here for CNS neuroprotection are also strongly implicated in anti-cancer applications^44^,^45^.

## Methods

### Reagents and subfractionation of PBI-05204

PBI-05204 was provided by Phoenix Biotechnology, Inc. (San Antonio, TX) and is an ethanol-modified supercritical CO_2_ extract of *Nerium oleander*^44^. Pure oleanolic acid was purchased from Sigma-Aldrich. Stock solutions were made in DMSO and diluted into brain slice culture medium to a final DMSO concentration of 0.1% for all conditions.

Subfractions of PBI-05204 were isolated by subjecting the whole supercritical CO_2_ extract of *N. oleander* to further extraction with hexane. The water soluble portion of that extract was further separated by reversed-phase chromatography (ODS) via sequential elution washes consisting of 30% (Fraction 0-H), 55% (Fraction 0–2), 80% (Fraction 0–3), 100% methanol (Fraction 0–4), and finally acetone-methanol (2:1 v:v; Fraction 0–5). All fractions were then subjected to analysis for relative content of oleandrin. The 100% methanol fraction was found to be free of all detectable oleandrin by HPLC analysis (Zorbax SB-18 column; Agilent). For confirmation, oleandrin levels in all five fractions were further analyzed using a more sensitive HPLC/MS method as previously described^46^.

In order to investigate its phytochemical composition, Fraction 0–4 (PBI-04711) was dissolved in a small quantity of methylene chloride. The insoluble part was composed mostly of triterpenoids based on the analysis of the ^1^H NMR and thin-layer chromatography visualized by sulfuric acid. The insoluble part (200 mg) was then subjected to a flash silica gel chromatograph and eluted into fractions using a gradient of CHCl_3_:MeOH (0% ~ 8%). Fraction 42 (146 mg) was repeatedly crystalized and found to consist of pure oleanolic acid (20 mg). The supernatants of the crystallization reactions were combined. A fraction of the supernatant (16.1 mg) was further purified by preparative HPLC using a Phenomenex Luna C18(2), 5 μm, 250 × 21.25 mm column, eluted with a gradient of MeOH:H_2_O 20 ~ 100% in 40 min, flow rate 9 mL/min to yield a mixture of oleanolic acid and ursolic acid (13.9 mg) as major components and a pure compound betulinic acid (0.3 mg) as a minor component. Another fraction of the supernatant was first separated by preparative-TLC to generate two fractions, which were further purified by preparative HPLC to yield two pure compounds uvalol (0.5 mg) and ursolic aldehyde (0.8 mg). Fraction 54 (22.6 mg) was repeatedly purified on HPLC column to obtain 3*β*,27-dihydroxy-12-ursen-28-oic acid (0.5 mg). Another fraction of the insoluble part (280 mg) was separated using a RediSep Rf Gold Silica Gel column (80 g). Pure ursolic acid (5.8 mg) was obtained from fraction 78 by repeated crystallization. Fraction 105 was purified by preparative HPLC to yield a mixture of *E*/*Z*-27-(*p*-coumaroyl)ursolic acid (2.2 mg). Efforts to separate these two compounds were not successful because of their rapid *cis*-*trans* isomerization. Based on the isolation procedure, the content of oleanolic acid in Fraction 0–4 was estimated to be ~35%, and that for the next two most abundant triterpenoids, ursolic acid and betulinic acid, to be ~25% and ~11%, respectively. The structures of these compounds were determined by 1D and 2D NMR and comparison with data in the literature.

To confirm the relative abundance of its major triterpenoid components, Fraction 0–4 was separated chromatographically and quantified against triterpene standards for oleanolic acid, ursolic acid, and betulinic acid (Sigma; see Supplementary Fig. S1).

### Brain slice assays for CNS injury and neurodegeneration

Coronal brain slices (250 μm thick) were prepared from postnatal day 10 Sprague-Dawley rat pups of either gender (Charles River) and established in organotypic culture as previously described^7^,^8^,^9^. All experimental procedures including the sacrificing of animals were done in accordance with NIH guidelines and under Duke IACUC approval and oversight. Briefly, brain tissue slices were cut in ice-cold artificial cerebrospinal fluid (ACSF) and plated in interface configuration on top of culture medium (Neurobasal A medium supplemented with 15% heat-inactivated horse serum, 10 mM KCl, 10 mM HEPES, 100 U/ml penicillin/streptomycin, 1 mM sodium pyruvate, and 1 mM L-glutamine) set in 0.5% reagent-grade agarose. To model ischemic injury, brain slices were subjected to oxygen-glucose deprivation (OGD) by exposure to glucose-free, N_2_-bubbled ACSF containing low O_2_ (<0.5%) for 5.5 min.

One h later, control and OGD-treated brain slices were biolistically transfected with DNAs encoding yellow fluorescent protein (YFP). For assays modeling neurodegeneration in AD or FTD, brain slices were co-transfected with YFP together with an expression construct for WT amyloid precursor protein (APP) as previously described^9^, or with YFP together with a cDNA constructed in-house encoding human tau4R0N (identical to NCBI Reference Sequence NM_016834), respectively. Brain slice explants were then incubated for 24 h under 5% CO_2_ at 37 °C for OGD assays; or for 3 d for APP- and tau4R0N-induced neurodegeneration assays. PBI-05204, subtractions thereof, and/or oleanolic acid were added to the culture medium at the time of brain slice explantation at the indicated concentrations.

For all brain slice assays, numbers of healthy pyramidal neurons in the cortical regions of each brain slice were imaged on a Leica MZIIIFL fluorescence stereomicroscope. Cortical pyramidal neurons were readily identified by their characteristic positions and orientations in the cortical plate, and by their prominent extension of a single, apical dendrite radially towards the pial surface. Healthy cortical pyramidal neurons were deemed as those 1) presenting a stout and brightly labeled cell body located within the pyramidal neuronal layers of the cortex; 2) retaining a clear apical dendrite extending radially towards the pial surface the slice; 3) expressing >2 clear basal dendrites >2 cell body diameters long directly from the neuronal soma; and 4) showing clear and continuous cytoplasmic labeling with the YFP visual marker in the soma and throughout all neuronal processes. Statistically significant differences with respect to the negative control condition (OGD, APP-transfected, or tau4R-transfected treated with DMSO carrier only) were determined using ANOVA followed by Dunnett’s *post hoc* comparison test at the 0.05 confidence level, with N = 12 brain slices per condition. Each experiment was carried out at least 3 times.

### Determination of Nrf2 activation and qPCR quantification of ARE gene expression

Primary corticostriatal neuronal co-cultures were prepared from E18 Sprague-Dawley rat or C57Bl/6 mouse embryos of either gender as previously described^16^. For luciferase reporter assays, the Cignal Antioxidant Response Reporter kit (Qiagen) was used. The 5xARE luciferase reporter mixture at 40:1 luciferase:*Renilla* plasmid was transfected into cortical and striatal neurons separately using an Amaxa electroporation device (Lonza). After electroporation, neurons were pooled and immediately plated into 96-well plates containing mature glial cultures. After culturing for 96 h, compounds were added at the indicated concentrations for 7 or 24 h prior to harvesting using Dual-Glo Luciferase Assay System protocol and reagents (Promega). Dual-wavelength luminescence was detected using a SpectraMax L microplate reader (Molecular Devices). Luciferase values were normalized to the internal *Renilla* control and fold-expression over the DMSO-only treatment control was calculated. At least 3 independent experiments were done using 4–6 biological replicates.

For qPCR quantification of ARE target gene expression levels, cortical and striatal neurons were plated onto 96-well plates containing mature glial cultures and cultured for 96 h. Fraction 0–4 was added to cultures at the indicated concentrations for 6 h. At the end of the treatment period, cells were lysed and total RNA was isolated using Absolutely RNA mini-prep kits (Agilent Technologies/Stratagene). cDNA was generated using oligo dT primers and Superscript II reverse transcriptase (Invitrogen). Resulting cDNA was used for quantitative PCR of gene transcripts using SYBR Green Real-Time PCR Master Mix (Life Technologies) and the following mouse primers, for: *Gclc* (forward-5′ TGGCCACTATCTGCCCAATT-3′ and reverse-5′- GTCTGACACGTAGCCTCGGTAA-3′), *Nqo1* (forward-5′-GCCCGCATGCAGATCCT-3′ and reverse 5′-GGTCTCCTCCCAGACGGTTT3′), *Srx* (forward-5′-GCTTCCTCTCGGGAGTCCTT-3′ and reverse-5′-CAGCAACAGCGACTACGAAGTAA-3′), and *Hmox1* (forward-5′-CCTCACTGGCAGGAAATCATC-3′ and reverse-5′-CCTCGTGGAGACGCTTTACATA-3′) (Integrated DNA Technologies). For rat corticostriatal co-culture samples, qPCR primers used were as previously described^47^. Each biological sample was measured in triplicate on a ViiA 7 real-time PCR instrument (Applied Biosystems); fold expression was calculated after normalization to corresponding control *GAPDH* levels.

## Additional Information

**How to cite this article**: Van Kanegan, M. J. *et al*. Dual activities of the anti-cancer drug candidate PBI-05204 provide neuroprotection in brain slice models for neurodegenerative diseases and stroke. *Sci. Rep.* **6**, 25626; doi: 10.1038/srep25626 (2016).

## Supplementary Material

Supplementary Information

## Figures and Tables

**Table 1:** Subfractionation of PBI-05204.

Name	Oleandrin	Other CGs
Fraction 0–H	No	No
Fraction 0–2	No	Yes
Fraction 0–3	Yes	Yes
Fraction 0–4	No	No
Fraction 0–5	No	No

Separation of the PBI-05204 botanical extract into subfractions containing or not containing oleandrin and/or other cardiac glycosides (CG). To identify neuroprotective constituents in addition to oleandrin, the PBI-05204 extract was chemically separated into 5 subfractions as described in the Methods. Each subfraction was then characterized as containing or not containing detectable amounts of oleandrin and/or other cardiac glycoside compounds as indicated in the table.
