# Supplementary material for: Dual activities of the anti-cancer drug candidate PBI-05204 provide neuroprotection in brain slice models for neurodegenerative diseases and stroke
Source: Sci Rep. 2016 May 12;6:25626. doi: 10.1038/srep25626 (PMC4865873; doi:10.1038/srep25626)

Supplementary Information

Dual activities of the anti-cancer drug candidate PBI-05204 provide neuroprotection in brain slice models for neurodegenerative diseases and stroke

Michael J. Van Kanegan, Denise E. Dunn, Linda S. Kaltenbach, Bijal Shah, Dong Ning He, Daniel D. McCoy, Peiying Yang, Jiangnan Peng, Li Shen, Lin Du, Robert H. Cichewicz, Robert A. Newman, and Donald C. Lo

Supplementary Figure S1. Relative abundance of the 3 major triterpenoid constituents of Fraction 0-4 as shown by chromatographic separation. A, Chromatogram of Fraction 0-4. **B,** Chromatograms of triterpene standards for oleanolic acid ("O"), ursolic acid ("U"), and betulinic acid ("B"; all purchased from Sigma) as indicated. Elution conditions were isocratic 95% MeCN with 0.1% formic acid at a flow rate of 0.6 mL/min using a Gemini C-18 column.

Supplementary Figure S1

A

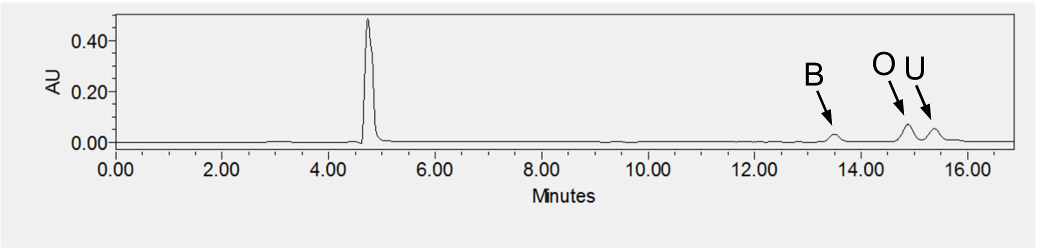

Fraction 0-4

B

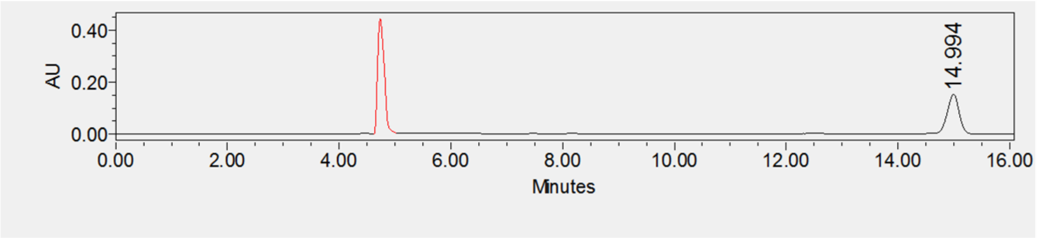

Oleanolic acid

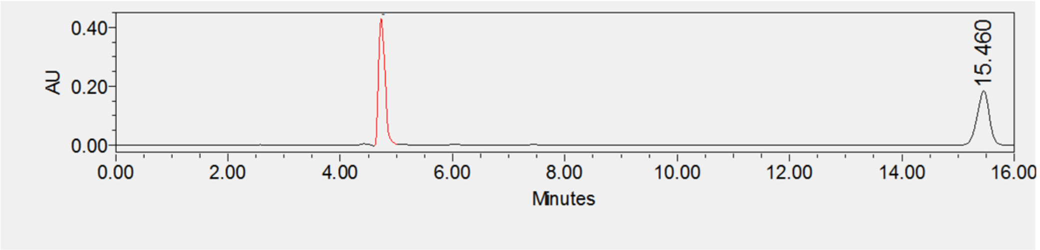

Ursolic acid

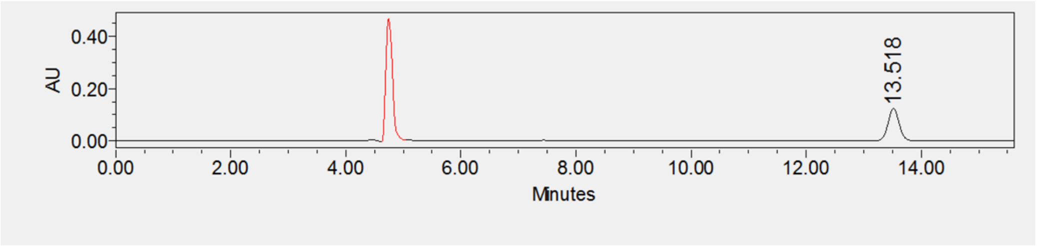

Betulinic acid

Supplementary Figure S2. Activation of ARE target genes by ursolic acid and betulinic acid. Activation of the ARE genes by ursolic acid and betulinic acid was examined using more closely-spaced concentration steps, showing that betulinic acid, like ursolic acid, is able to induce clear upregulation of *Srx* and *Hmox1* despite its toxicity at higher concentrations (see Fig. 5). Rat primary corticostriatal co-cultures were treated for 6 h with Fraction 0-4 (in µg/ml) or ursolic acid and betulinic acid (in µM) at the concentrations indicated, then harvested and processed for qPCR analysis of the ARE target genes shown. Quantitative RNA values were normalized to the *GAPDH* reference control and fold-expression changes are expressed relative to the DMSO-carrier only condition ("--") set to a value of 1. Dark blue bars denote statistically significant differences with respect to the DMSO-carrier only control by a Student's *t*-test at $p<0.05$.

Supplementary Figure S2

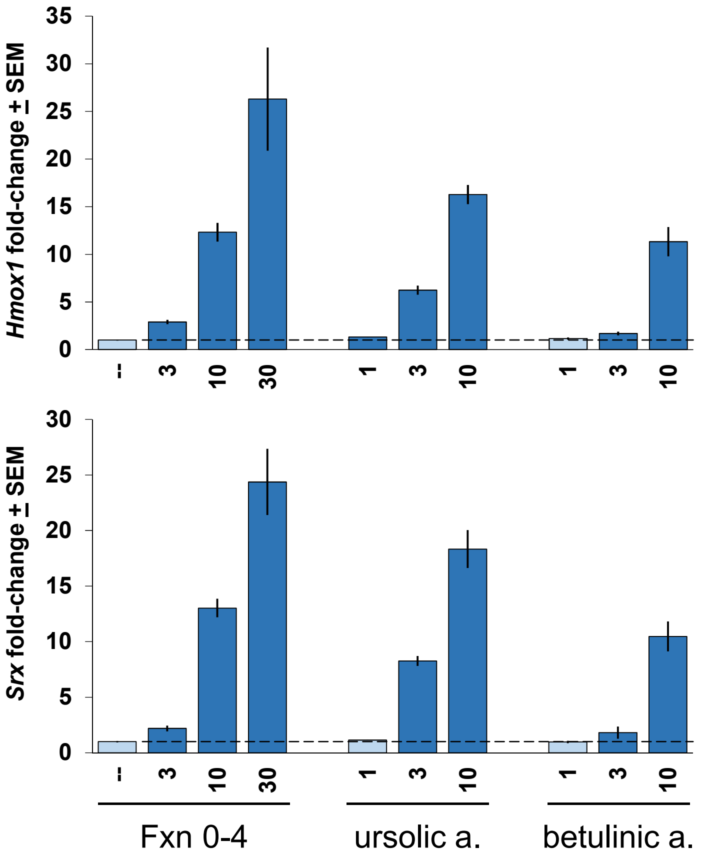

Supplement: Supplementary Information [file srep25626-s1.pdf]
